# A practical approach to the clinical challenges in initiation of basal insulin therapy in people with type 2 diabetes

**DOI:** 10.1002/dmrr.3418

**Published:** 2020-12-01

**Authors:** Thomas Forst, Pratik Choudhary, Doron Schneider, Bruno Linetzky, Paolo Pozzilli

**Affiliations:** ^1^ Department of Endocrinology and Metabolic Diseases Universitatsmedizin der Johannes Gutenberg Mainz Germany; ^2^ Clinical Research Services Manhheim Germany; ^3^ Department of Diabetes and Nutritional Sciences King's College London UK; ^4^ Tandigm Health Conshohocken Pennsylvania USA; ^5^ Eli Lilly and Company Buenos Aires Argentina; ^6^ Department of Endocrinology and Metabolic Diseases Università Campus Bio‐Medico Rome Italy; ^7^ Centre of Immunobiology Barts and the London School of Medicine Queen Mary University of London UK

**Keywords:** basal insulin, initiation, type 2 diabetes

## Abstract

Initiating insulin therapy with a basal insulin analogue has become a standard of care in the treatment of type 2 diabetes mellitus (T2DM). Despite increasing choices in pharmacological approaches, intensified glucose monitoring and improvements in quality of care, many patients do not achieve the desired level of glycaemic control. Although insulin therapy, when optimized, can help patients reach their glycaemic goals, there are barriers to treatment initiation on both the side of the patient and provider. Providers experience barriers based on their perceptions of patients' capabilities and concerns. They may lack the confidence to solve the practical problems of insulin therapy and avoid decisions they perceive as risky for their patients. In this study, we review recommendations for basal insulin initiation, focussing on glycaemic targets, titration, monitoring, and combination therapy with non‐insulin anti‐hyperglycaemic medications. We provide practical advice on how to address some of the key problems encountered in everyday clinical practice and give recommendations where there are gaps in knowledge or guidelines. We also discuss common challenges faced by people with T2DM, such as weight gain and hypoglycaemia, and how providers can address and overcome them.

## 1 INTRODUCTION

Despite the availability of a wide range of glucose‐lowering therapies many patients with type 2 diabetes mellitus (T2DM) need insulin therapy because of progressive pancreatic β‐cell failure.^1^, ^2^, ^3^, ^4^ The introduction of newer oral and injectable therapies, some with favourable cardiovascular profiles or with other benefits such as low risk of hypoglycaemia or weight loss,^2^ may delay initiation of insulin treatment to later stages of T2DM than before. However, insulin, often in combination with other medications, remains a necessary treatment for many patients in whom depletion of endogenous insulin secretion can no longer be compensated for.

The introduction of basal insulin analogues has changed the treatment paradigm for T2DM.^5^, ^6^, ^7^ Initiating insulin therapy with a basal insulin analogue has become a standard of care in T2DM.^8^, ^9^ The simplicity of therapy, ease of dose adjustment and blood glucose monitoring, the low relative risk of hypoglycaemia, and limited weight gain, has increased the confidence of non‐endocrinologists to make prescribing decisions and today basal insulin treatment is often initiated by general practitioners and nurses.^10^, ^11^ However, despite increasing access to basal insulin analogues, glucose monitoring devices, and improvements in quality of care, many patients do not achieve the desired level of glycaemic control.^12^, ^13^, ^14^

Barriers to the achievement of desired glycaemic control include those on both the patients' as well as provider's part. Many patients are reluctant to start insulin therapy or stop insulin therapy soon after initiation because of concerns, fears, or lack of perceived need for insulin treatment.^9^, ^15^, ^16^, ^17^ Among those who continue therapy, many use suboptimal doses.^13^, ^18^ On the other hand, clinicians may lack confidence in their ability to reassure patients or solve the practical problems of insulin therapy and avoid decisions they deem risky for their patients (e.g., increasing dose).^19^, ^20^, ^21^, ^22^

Guidelines provide general guidance for insulin initiation, however, they do not answer practical questions and or address everyday challenges faced by clinicians. For example, guidelines give recommendations on initial insulin dose and its titration, but in the real world, insulin requirements in different patients with T2DM may vary widely due to individual circumstances such as glycaemic control, comorbidities, concomitant medications, adverse events, patient preferences, and cost concerns.

**FIGURE 1:**
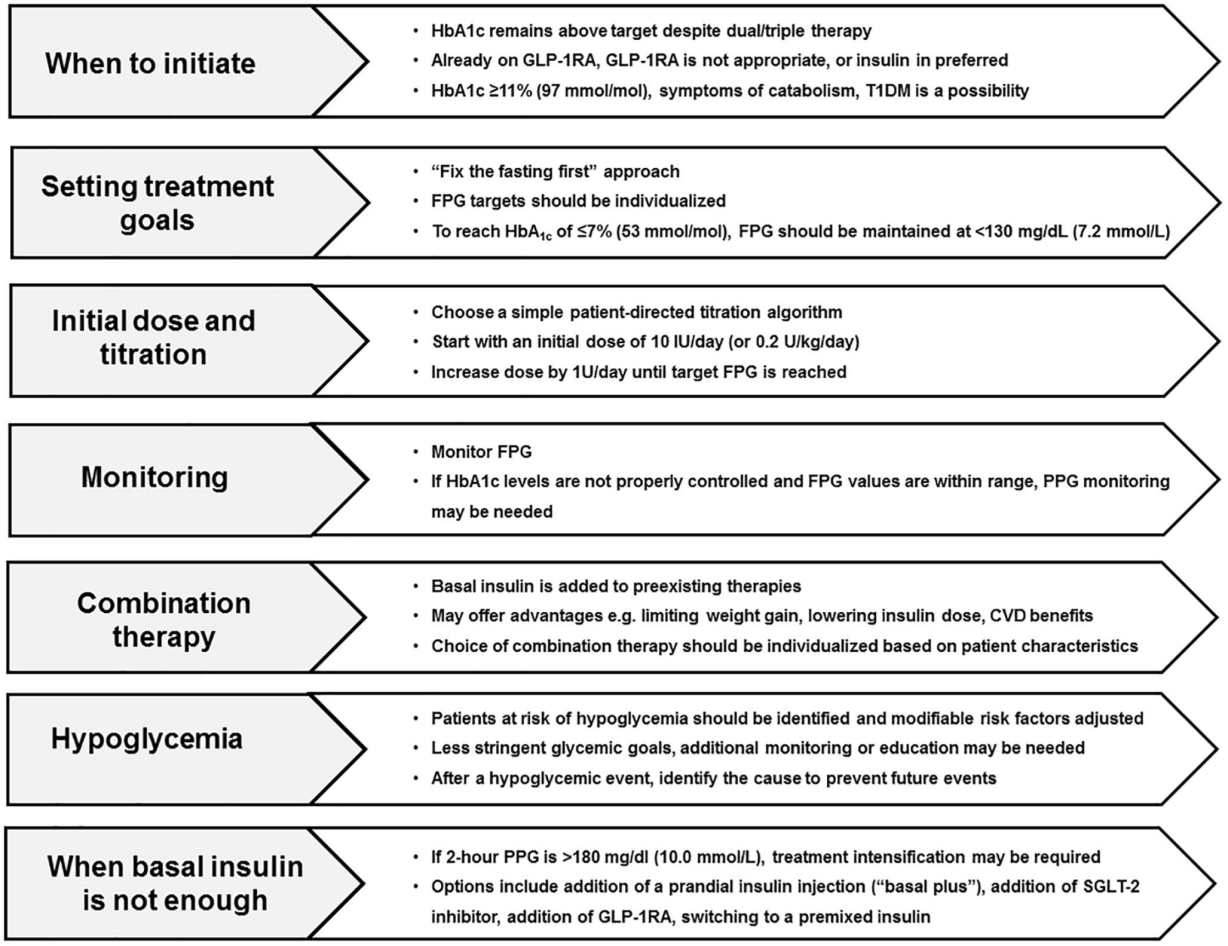
Key considerations when initiating insulin therapy. CVD, cardiovascular disease; FPG, fasting plasma glucose; GLP‐1RA, glucagon‐like peptide‐1 receptor agonist; HbA1c, haemoglobin A1c; PPG, postprandial glucose; SGLT‐2, sodium–glucose cotransporter‐2; T1DM, type 1 diabetes mellitus

In this study, we provide practical advice on how to address some of the problems encountered by clinicians in daily clinical practice. While addressing emotional aspects of insulin initiation is fundamental for successful treatment in T2DM, in this opinion study, we focus on the practical aspects related to the use of insulin. An overview of key considerations for initiating insulin therapy is provided in Figure 1.

### 1.1 When should insulin be initiated?

The decision to start insulin therapy is straightforward in patients with very poor glycaemic control, for example, blood glucose exceeding 300 mg/dl (16.7 mmol/L) or HbA1c >11% (97 mmol/mol) and in those with symptomatic hyperglycaemia or with evidence of catabolic state as reflected by weight loss or ketosis.^2^, ^8^ There is a consensus that insulin should be started right away irrespective of previous therapy in these situations.^2^ In patients whose metabolic control has deteriorated quickly, in whom other non‐insulin treatments were ineffective and who had low or normal body weight or experienced weight loss, the possibility of type 1 diabetes mellitus or latent autoimmune diabetes in adults (LADA) as an underlying cause of metabolic decompensation should be considered and insulin started promptly.^2^, ^4^ The diagnosis of LADA is confirmed with the presence of islet autoantibodies such as glutamic acid decarboxylase antibodies and by C‐peptide measurements.^23^, ^24^ Due to the heterogeneity of LADA, a personalized treatment approach is required.^23^, ^25^

Insulin remains the pharmacological treatment of choice in pregnant women with T2DM or gestational diabetes; however, these patients should be referred to speciality centres and require multiple daily injections of insulin,^26^ and thus are not within scope for this study.

More frequently, making a decision to start insulin therapy is less straightforward. Insulin initiation is generally recommended when blood glucose concentrations cannot be controlled with diet and lifestyle modifications alone or in combination with dual/triple non‐insulin therapies.^2^, ^4^ Apart from the efficacy of insulin where other therapies turn out to be ineffective, benefits of insulin include possible reductions in the number of drugs required for glucose control.

Clinicians may wonder which of the injectable therapies should be used first: basal insulin or glucagon‐like peptide‐1 receptor agonists (GLP‐1 RAs). Because several of the GLP‐1 RAs have been shown to reduce the risk of cardiovascular disease (CVD) in T2DM, and they typically do not increase the risk of adverse events commonly seen with insulin therapy such as weight gain and hypoglycaemia, they are recommended as the first injectable option in patients with CVD, heart failure, or chronic kidney disease, or in patients with a compelling need to reduce risk of hypoglycaemia; and for patients in whom weight gain maybe a concern.^2^ Insulin may be preferable as the first injectable treatment in other patients, especially those with a rapid decline in β‐cell function, contraindications to GLP‐1 RAs (e.g., history of pancreatitis or severe gastrointestinal diseases), and low tolerance of gastrointestinal side effects.^2^, ^27^ Of note, treatment with insulin and GLP‐1 RA can be started simultaneously,^2^ especially in patients with severely deranged glycaemic control (e.g., HbA1c >10% [86 mmol/mol]). The choice between GLP‐1RAs and basal insulin as the first injectable therapy in T2DM is not only a matter of safety but also of efficacy, as some once‐weekly GLP‐1RAs have demonstrated significant HbA1c reductions when compared to basal insulins.^28^, ^29^ In some health systems, insulin is used as the first injectable based on cost.

There are other situations when starting insulin therapy is appropriate irrespective of other/previous treatments. These include periods of metabolic decompensation due to the inter‐current illnesses, glucocorticoid treatment, or scheduled surgery.^30^, ^31^, ^32^ Even though insulin therapy is typically instituted for a few days or weeks in these circumstances, such a short course of treatment may reduce glucotoxicity, improve β‐cell function, and metabolic control in the longer term.^33^ These situations go beyond the scope of this review and may require not only basal insulin use, but also rapid‐acting insulins.

## 2 SETTING THE GOALS OF THERAPY

Initiation of insulin therapy should be an opportunity to revise glycaemic targets and HbA1c goals. Goals from the period prior to insulin therapy might continue to be appropriate. However, as insulin increases the potential risk of hypoglycaemia, setting up less stringent targets for patients at risk of hypoglycaemia or who are otherwise vulnerable may be considered.

In patients at high risk of hypoglycaemia, fasting glucose targets can be incrementally reduced as patients learn the principles of diabetes self‐management education and support^34^ to help them reach the most appropriate targets.

Introduction of a once‐daily injection of basal insulin allows simple blood glucose monitoring to adjust insulin dose and monitor progress of therapy focussing primarily on fasting plasma glucose (FPG) values (‘fix the fasting first’ approach).^35^, ^36^ FPG targets should therefore be optimized first to guide treatment adjustments. Table 1 provides average FPG values for a range of specified HbA1c targets of 5.5%–8.5% (37–69 mmol/mol) in patients with T2DM based on empirical data.

**TABLE 1:** Average FPG values for achieving a range of HbA1c targets in type 2 diabetes mellitus^a^

	**HbA1c group, % (mmol/mol)**				
	5.5–6.49 (37–47)	6.5–6.99 (47–53)	7.0–7.49 (53–58)	7.5–7.99 (58–64)	8.0–8.5 (64–69)
	**Estimated average glucose, mg/dl**				
	111–139	140–153	154–168	169–182	183–197
Mean FPG, mg/dl (95% CI)	122 (118–127)	139 (139–147)	147 (133–161)	157 (139–176)	179 (158–201)

Abbreviations: CI, confidence interval; FPG, fasting plasma glucose; HbA1c, haemoglobin A1c.

^a^ Modified from Wei et al., 2014.^94^

If the goal of HbA1c ≤ 7% (53 mmol/mol) is adopted, FPG should be maintained at <130 mg/dl (7.2 mmol/L) and in the range of 80–130 mg/dl (4.4–7.2 mmol/L).^30^ In patients for whom hypoglycaemia is a concern, FPG should be maintained at ≥100 mg/dl (5.6 mmol/L)^37^ based on the ‘Treat‐to‐Target’ paradigm of dose adjustments to make insulin therapy simpler for patients and clinicians alike. More aggressive FPG targets may be adopted for patients requiring more stringent glycaemic control and those who are able to manage the therapy without increasing the risk of hypoglycaemia.

## 3 INITIAL DOSE AND TITRATION

When choosing an initial dose of insulin both the provider and the patient should realize this starting dose will almost always not be enough. The typical approach is to commence with a lower dose and increase to meet glycaemic targets.^39^, ^40^ In most patients, either a fixed dose of 10 U per day is used or the dose is calculated based on body weight with 0.1–0.2 units/kg/day.^8^ ^,^ ^40^ ^,^ ^41^ In cases of severe hyperglycaemia with evidence of catabolism, an even larger starting dose of 0.3–0.4 units/kg/day may be required.^42^ Patients should understand the titration process, should be aware a higher dose of basal insulin may be required to achieve their target,^38^ and that it takes an average of 8–12 weeks to reach the fully effective dose.^39^ In a pooled analysis of 22 randomized controlled trials of patients with T2DM initiating basal insulin (insulin glargine or insulin detemir), the mean HbA1c reduction at endpoint was 1.4% (15 mmol/mol) and insulin dose ranged from 23 to 71 units.^43^

It is a good practice to inject the first dose in the provider's office to ensure the patient understands the practical aspects of dose dialling and proper administration.^44^ Starting at a lower dose and gradually increasing, it reduces the risk of hypoglycaemia in the early period of therapy, provides time for incorporation of insulin therapy into daily routine, time to adjust concomitant treatments, and time for additional education. Access to diabetes education is important: the patient could be referred to a certified diabetes educator (CDE) or a diabetes education centre outside of the office. A team‐based approach to the care of patients with T2DM has been shown to improve health outcomes.^45^

Many titration algorithms are available including those from world medical organizations and those reported in clinical trials. The simplest algorithm for both patient and healthcare provider should be chosen and should be based on individual needs and lifestyle. An example of a simple patient‐directed titration algorithm is to start with an initial dose of 10 U/day and increase the dose by 1 U per day until the target FPG is reached. The use of simpler algorithms may also help tackle titration inertia which is a common problem in T2DM following insulin initiation.^22^

Titration can be performed by the healthcare provider or the patient. At the initiation of treatment, it should be decided whether the patient can self‐titrate or not. Patient‐led titration regimens are preferable and have been shown to be efficacious in achieving glycaemic control with a low risk of hypoglycaemia.^46^, ^47^ To initiate titration, the healthcare provider ideally should generate a pre‐printed algorithm for the patients.

Before initiating insulin therapy, the physician should ensure the patient will be able to monitor blood glucose levels, is aware of hypoglycaemia symptoms, and knows how to deal with hypoglycaemic episodes. The patient should be trained how to use blood glucose values to adjust food intake, exercise, and the dose in order to achieve glycaemic goals and avoid hypoglycaemia and should also be directed how to report the results to their professional healthcare team in an effective and timely manner. This education and support may be more efficiently and effectively provided by a non‐physician provider such as a CDE. The official consensus statement of the American Association of Clinical Endocrinologists and American College of Endocrinology recommends some methods of communicating the results to the healthcare team, including using a logbook at the time of the office visit, computer outputs (graphs, statistics), periodic phone calls/emails, automated transfer from metre or sensor to Internet for review, or automated interpretation by glucose monitoring device.^48^

## 4 MONITORING

### 4.1 How to monitor

If the patient is receiving only basal insulin, with or without other diabetes medications, blood glucose monitoring should be performed when fasting to evaluate the impact of basal insulin on overnight glycaemic control.^48^ If HbA1c levels are not properly controlled and FPG values are within range, additional monitoring may help evaluate postprandial glucose (PPG) control to decide if treatment intensification is needed. PPG monitoring does not need to occur after each meal and can be rotated or performed after the largest meal. Additional monitoring may also help the patient understand glycaemic fluctuations which may aid in identifying situations that can increase the risk of hypoglycaemia; for example, a large decline in bedtime to morning blood glucose may herald overlooked nocturnal hypoglycaemia.

In recent years, continuous glucose monitoring (CGM) has emerged as a complementary method for the assessment of glucose levels.^49^ Although there is no evidence for use of CGM in patients with T2DM initiating basal insulin, it may be useful in the case of patients intensifying insulin therapy with prandial insulin.

### 4.2 Glycaemic variability

Glycaemic variability is a term used ambiguously to describe both within‐day and inter‐day variations in blood glucose values. Within‐day blood glucose fluctuations are normal and represent glucose fluctuations caused by food and exercise. Given the recommendation to monitor only FPG in patients using basal insulin, inter‐day glucose variations are a more frequent and relevant problem in this type of patient.

Glucose variability is a predictor of hypoglycaemia.^50^, ^51^ High glucose variability in patients with T2DM is associated with increased rates of overall symptomatic, nocturnal symptomatic and severe hypoglycaemia,^52^ and needs to be addressed in order to mitigate the risk.

There are many factors that may affect glucose variability and identifying them can help both the healthcare provider and patient to understand the clinical scenarios and make the appropriate decisions. Evidence suggests stressful experiences may destabilize blood glucose levels through physiological mechanisms but also through interfering in self‐care behaviours.^53^ A diabetes‐specific instrument, where patients look for patterns in their perceived stress levels and their blood glucose levels, may help patients to understand the relationship between stress and blood glucose levels.^53^ A higher degree of physical activity can lower FPG,^54^ and therefore, a change in exercise pattern could cause variability in FPG. Specific exercise recommendations are discussed in a later section. Dietary changes can impact FPG variability. For example, a very low‐carbohydrate diet or alcohol consumption can affect insulin sensitivity and blood glucose fluctuations.^55^, ^56^ Avoiding extreme changes in diet and limiting alcohol intake are thus recommended to reduce FPG variability.

Improper insulin injection technique may also be a factor in glucose variability.^57^, ^58^ To avoid insulin leakage and ensure the full dose has been delivered after the insulin has been injected, patients should count slowly to 5 or 10, then withdraw the needle from the skin.^59^, ^60^ As discussed later, patients should also practice injection site rotation to prevent lipo‐hypertrophy, which can impact the consistent delivery of the full intended dosage of insulin, and thus contribute to glucose variability.^57^

Postprandial hyperglycaemia is also one of the earliest abnormalities of glucose homoeostasis associated with T2DM^61^ and a contributor to glycaemic variability^62^ and overall glycaemic control. The recommendation is to focus on FPG and not PPG to simplify the monitoring schedule and in the expectation that normalization of FPG will also lower PPG. However, occasionally measuring blood glucose after meals (e.g., after the largest meal or after specific foods) may give the patient and provider a more complete picture and can increase patients' awareness of the relationship between their lifestyle and daily glycaemic fluctuations, encouraging patients to engage in behaviours benefitting glycaemic control.^62^ When examining within‐day glucose variability, the primary focus should be on the low blood glucose values. Additional monitoring may be warranted on some occasions to rule out the occurrence of hypoglycaemia. Blood glucose levels may need to be checked more frequently, and insulin dose adjusted, until glucose variability decreases. The use of a Flash glucose monitoring system, or other CGM systems, may be helpful for analysing glucose variability.

### 4.3 Combining non‐insulin anti‐hyperglycaemic medications with insulin

A variety of oral and injectable anti‐hyperglycaemic treatments are available for the treatment of T2DM and some can be combined.^2^ Combination therapy allows for the use of medicines that have synergistic effects by addressing different pathophysiological alterations in T2DM.^63^ Combination therapy of insulin with other agents may also offer a number of advantages such as limiting weight gain and lowering insulin dose (e.g., in combination with metformin, sodium–glucose cotransporter‐2 [SGLT‐2] inhibitor and GLP‐1RAs), and some medications also exert favourable cardiovascular effects.^2^ ^,^ ^27^ ^,^ ^64^

The choice of combination therapy should be individualized based on patient characteristics and careful consideration of the advantages and disadvantages of anti‐hyperglycaemic agents. Many factors such as age of the patient, risk of hypoglycaemia, comorbidities, presence of complications, risk of weight gain after insulin initiation, cost of treatment, and so on should be taken into account when tailoring combination therapy to individual patient needs.

In general, basal insulin is added to pre‐existing therapies with some specific exceptions. Best practices for combining insulin with various classes of non‐insulin anti‐hyperglycaemic medications are summarized in Table 2.

**TABLE 2:** Best practices when combining insulin with non‐insulin anti‐hyperglycaemic treatments

Drug class/name	Best practice
Sulfonylureas	Stop sulfonylureas when initiating basal insulin due to hypoglycaemia risk^67^
Thiazolidinediones	Stop thiazolidinediones or reduce dose when initiating basal insulin^2^
Metformin	Continue treatment with metformin when initiating basal insulin^2^, ^65^
GLP‐1RAs	Combining GLP‐1 RAs with basal insulin has high efficacy and limits weight gain and hypoglycaemia; consider GLP‐1 RAs if a patient has established cardiovascular disease or chronic kidney disease^2^
DPP‐4 inhibitors	Do not combine insulin with DPP‐4 inhibitors if a patient is also using a GLP‐1 RA^2^
SGLT‐2 inhibitor	Consider a SGLT‐2 inhibitor if patient has established cardiovascular disease, chronic kidney disease, or heart failure; insulin dose may need to be reduced to prevent hypoglycaemia^2^

Abbreviations: DPP‐4, dipeptidyl peptidase 4; GLP‐1RAs, glucagon‐like peptide‐1 receptor agonists; SGLT‐2, sodium–glucose cotransporter‐2.

Metformin remains the first line of therapy for most patients with T2DM who can tolerate it and do not have contraindications.^2^ Most patients with T2DM starting insulin will be using metformin as a background treatment. It is a good practice to continue metformin when initiating insulin as by reducing insulin resistance, metformin may reduce insulin dose requirements and prevent weight gain.^2^ ^,^ ^65^ ^,^ ^66^

For patients initiating insulin who are currently taking a sulfonylurea (SU), the recommended best practice is to stop SU treatment to avoid risk of hypoglycemia.^67^

Another class of drugs which may raise the risk of AEs when combined with insulin are thiazolidinediones because of their risk of oedema and heart failure.^2^ Caution is warranted and thiazolidinediones should be discontinued in patients at risk, for example, patients with pre‐existing heart failure and older patients.^2^

Other drugs which are commonly used prior to use of basal insulin include SGLT‐2 inhibitors and GLP‐1 RAs,^68^ ^,^ ^69^ particularly in patients with CVD, heart failure, or chronic kidney disease.^2^ Treatment with SGLT‐2 inhibitors and GLP‐1 RAs should continue after basal insulin initiation not only because of their complementary actions, their ability to prevent weight gain, and their ability to reduce insulin dose,^2^ ^,^ ^27^ ^,^ ^70^ but also because select SGLT‐2 inhibitors and GLP‐1 RAs have been shown to lower CVD risk in patients with atherosclerotic CVD.^2^ When insulin is added to SGLT‐2 inhibitors and GLP‐1 RAs, more cautious titration and monitoring may be warranted to limit hypoglycaemia risk with improved glycaemic control. Other recommendations (e.g., ketone monitoring to avoid diabetic ketoacidosis with SGLT‐2 inhibitors) are similar to those for patients taking these medications without insulin.^2^ Dipeptidyl peptidase 4 inhibitor treatment can be maintained in a patient initiating basal insulin unless they are used in combination with GLP‐1RA.^2^ ^,^ ^38^

As stated previously, initiation of basal insulin, which is among the most potent of glucose‐lowering medications, may be an opportunity to stop some previously used medications and to lower the burden of polypharmacy without sacrificing glycaemic control. Such a decision should be made after consideration of individual patient needs.

## 5 HYPOGLYCAEMIA

### 5.1 Incidence and risk factors

The incidence of hypoglycaemia in patients with T2DM initiating basal insulin is relatively low. It is estimated that less than 10% of patients with T2DM initiating basal insulin experience hypoglycaemia after 1–2 years of follow‐up.^71^, ^72^ The incidence of severe hypoglycaemia in these patients is even lower^73^, ^74^: less than 1% up to 4 years follow‐up.^75^, ^76^

Fear of hypoglycaemia is common and associated with discontinuation of insulin therapy even in patients who have not actually experienced hypoglycaemia.^77^ Understanding and addressing patients' feelings and concerns about hypoglycaemia is critical for successful treatment. It is important for patients to understand the potential risk factors for hypoglycaemia. Some of the common causes of hypoglycaemia are listed in Table 3.

**TABLE 3:** Common causes of hypoglycaemia

Change in diet^80^
Exercise^80^ ^,^ ^88^
Illness^89^
Titrating to lower blood glucose targets^95^ ^,^ ^96^
Inappropriate insulin use^80^
Physical or cognitive limitations^38^

Patients at risk of hypoglycaemia should be identified and modifiable risk factors should be adjusted (e.g., discontinuation of SU therapy). Risk factors for developing severe hypoglycaemia include comorbidities (e.g., renal function impairment), diabetes duration, advanced age, history of severe hypoglycaemia, high glycaemic variability, microvascular complications, and SU use.^50^ ^,^ ^78^ ^,^ ^79^ In such patients, less stringent glycaemic goals may need to be set and some may need additional monitoring or education.

### 5.2 What guidance should be provided for recognizing and treating hypoglycaemia?

Patients and their family members/caregivers should be educated on the signs and symptoms of hypoglycaemia. Hypoglycaemia can occur quickly, and people can experience different symptoms. The advice given to patients and their family members/caregivers should empower them to recognize and address hypoglycaemia effectively without scaring them. Understanding and addressing feelings and concerns about hypoglycaemia before and during insulin treatment is critical. If experiencing symptoms of hypoglycaemia, patients should measure their blood glucose if possible, follow the ‘15–15 rule’ and contact their healthcare provider (See Table 4).^80^ Where appropriate, care providers should be educated in rescue treatments such as oral glucose gels and intramuscular or nasal glucagon. Glucagon should be prescribed for all individuals at increased risk of level 2 hypoglycaemia, defined as blood glucose <54 mg/dl (3.0 mmol/L), to ensure availability in emergency situations.^30^

**TABLE 4:** The ‘15–15’ rule^a^

1. Consume 15 g of carbohydrates (e.g., ½ cup of juice).
2. After 15 min, check blood glucose.
3. If blood glucose is < 70 mg/dl (<3.9 mmol/L), consume another 15 g of carbohydrates.
4. Repeat until blood glucose is ≥ 70 mg/dl (≥3.9 mmol/L).
5. Eat a meal or snack to ensure blood glucose does not drop again.

^a^ Modified from American Diabetes Association.^80^

### 5.3 What steps should be taken following an episode of hypoglycaemia?

It is critical to understand what may have induced the hypoglycaemic episode and how future episodes can be prevented. Table 5 lists steps to be considered when evaluating the cause of hypoglycaemia. Preventive measures should be considered in high risk patients such as always carrying a source of rapid‐acting carbohydrate whenever leaving home.m

**TABLE 5:** Recommended actions following an episode of hypoglycaemia

1. Evaluate what happened.
Ask patients what happened on the day hypoglycaemia occurred to determine a potential cause.
Encourage additional SMBG measurements, as one low FPG reading may not be a sufficient reason to discontinue titration.
2. Discuss with the patient.
Understand that patients might be scared and discouraged.
Reassure patients that hypoglycaemia is usually rare and manageable.^38^
Talk about relevant potential causes of hypoglycaemia.^38^
3. Consider changes in insulin dose only after thorough evaluation and discussion to rule out preventable causes of hypoglycaemia.
A dose reduction of 2–4 U or 10%–20% has been proposed if no cause is identified.^38^
Patients may ultimately need a higher dose than the dose that was taken when they experienced the hypoglycaemic event.

Abbreviations: FPG, fasting plasma glucose; SMBG, self‐monitored blood glucose.

## 6 OTHER COMMON CLINICAL PROBLEMS AND QUESTIONS

### 6.1 What if despite adequate titration the patient's HbA1c is still high but FPG levels are within the recommended range?

Before adjusting medications, understand potential reasons for a disconnect between HbA1c and FPG values (e.g., glycaemic variability). If HbA1c is still higher than the target after 3–6 months but FPG is in the target range, have the patient measure PPG, as postprandial hyperglycaemia contributes to elevated HbA1c levels.^30^ If PPG values at 2 h are above range (>180 mg/dl [10.0 mmol/L]), there are several options.

The simplest manner of advancing therapy is by adding a prandial insulin injection to the basal regimen (‘basal plus’).^47^ ^,^ ^81^ One injection should be given with the largest meal of the day starting with a dose of either 4 units or 10% of the basal dose; increase the dose by 1–2 U or 10%–15% twice weekly).^2^, ^8^

Another option is to add a SGLT‐2 inhibitor if the patient is not already on it. When initiating a SGLT‐2 inhibitor, consider a lower dose of insulin to reduce hypoglycaemia risk.^2^ SGLT‐2 inhibitors have favourable impacts on weight and blood pressure, with low risk of hypoglycaemia.^2^ For patients with chronic kidney disease or atherosclerotic CVD, an SGLT‐2 inhibitor with proven benefit is recommended.^2^

A further option is to add a GLP‐1 RA to the basal regimen. GLP‐1 RAs can promote weight loss and have minimal risk of hypoglycaemia, but cost may be a consideration.^2^ When initiating a GLP‐1RA, a lower dose of insulin may be needed to reduce hypoglycaemia risk.^82^ ^,^ ^83^ For patients with atherosclerotic CVD, a GLP‐1RA with proven cardiovascular benefit is recommended.^2^ Fixed‐dose combinations of GLP‐1RAs and basal insulin analogues are available.^2^

Finally, the patient may switch to a premixed insulin.^2^ In this case, the basal insulin should be stopped and replaced by a twice or three times daily premix insulin regimen using the same total dose the patient was using prior to premixed.^84^ Use of premixed insulin is a simple, convenient means of spreading insulin across the day, particularly for patients who eat on regular schedules.^8^

### 6.2 What top messages should be reinforced to patients regarding needles and the actual injection?

Some tips for making injections less painful include using shorter needles (4, 5, or 6 mm), keeping the insulin in use at room temperature, injecting only when the alcohol has fully dried, avoiding injecting at hair roots, and using a new needle for every injection.^85^ Injection sites should be rotated and patients should be taught an easy‐to‐follow rotation scheme from the onset of insulin therapy.^86^ Injection sites should be inspected at every visit and patients should be taught how to inspect them on their own and how to detect lipo‐hypertrophy.^86^ Areas of lipo‐hypertrophy should be avoided for injection until the abnormal tissue heals.^86^

### 6.3 What advice should be given to patients when performing sports or exercising?

Insulin dose and carbohydrate intake changes may be needed to prevent exercise‐related hypoglycaemia.^87^ The risk of nocturnal hypoglycaemia following physical activity may be mitigated by reducing basal insulin dose, by including bedtime snacks, and/or by the use of continuous glucose monitoring.^87^ Conversely, individuals with T2DM may also experience hyperglycaemia after aerobic or resistance exercise and administer too little insulin for meals before activity.^87^

As a general rule, blood glucose levels should be checked before and after exercise. If blood glucose is ≤ 100 mg/dl (5.6 mmol/L) during or after exercise, a small snack before exercising or an adjustment in insulin may be necessary.^88^ The challenges related to blood glucose management with exercise vary with age, activity type, and the presence of diabetes‐related complications.^87^ Therefore, specific recommendations should be tailored to meet the needs of each patient.^87^

### 6.4 What instructions should be given to the patient who is travelling overseas?

Travel increases the risk of hypoglycaemia and can cause dehydration, especially if many time zones are crossed. Both stress during travel and decreased physical activity and enlarged food consumption often result in increased blood glucose levels.^89^ Patients should discuss plans with their diabetes team well in advance of any long‐distance travel. During travel, patients should carry their medical identification document, medical monitoring equipment, glucose medication, and any long‐acting carbohydrate snacks. For short duration trips (≤5 time zones or <3 days duration), patients should keep watches set to their local home time and continue to administer their basal insulin as usual.^90^ For longer duration travel (≥5 time zones or >3 days duration), adjustments in both timing and dosage of basal insulin are required.^90^

If the patient is traveling across 5 or more time zones and staying in a different time zone longer than 3 days and taking long‐acting basal insulin, travel requires a 4% adjustment to the insulin dose for each time zone traversed (1 h is 4% of the 24 h day), with westward travel resulting in missed doses and eastward travel resulting in excess doses.^90^ Guidelines, patient handouts, and dosage calculators for adjusting insulin regimens during travel are available.^90^ ^,^ ^91^

### 6.5 What should be done when a patient is gaining too much weight?

Weight gain may occur with initiation of basal insulin therapy and patient expectations in this regard need to be managed when basal insulin treatment is needed. Fear of insulin‐related weight gain can be a major barrier to the initiation and intensification of insulin therapy^92^ and can result in patients stopping insulin even if they have not experienced weight gain.^77^ Clinical trials have shown a modest increase in body weight of about 1 kg following insulin initiation.^65^ ^,^ ^93^ Addressing patient concerns about insulin‐related weight gain early in the course of treatment is important.

While diet plays an important role, adherence to a prescribed diet is commonly erratic in clinical practice, even with support of nutritionists and diabetes educators.^92^ Patients should be encouraged to limit mild, but frequent, defensive snacking, which can lead to weight gain.^92^ Nutritional education and the inclusion of a nutritionist as part of a patient's team‐based care may help counteract insulin‐induced weight gain.

Concomitant anti‐hyperglycaemic therapies can help with weight control by mitigating associated weight gain due to insulin or can even facilitate weight loss.^2^ Metformin should be continued to minimize weight gain and SGLT‐2 inhibitors and GLP‐1 RAs can help to avoid insulin‐associated weight gain or even reduce body weight.^2^, ^67^

Discussing and explaining some of the factors or processes behind insulin‐related weight gain may ease patient concerns. One factor may be ‘catch‐up’ weight gain which is the regain of uncontrolled diabetes‐induced weight loss (e.g., in patients who were previously in a catabolic state or had very high blood glucose values).^92^ Another factor is the fact that insulin is a hormone and can stimulate the formation of fat.^92^ Other risk factors or causes of weight gain such as fear of hypoglycaemia or treatment of depression should also be explored and addressed. A gradual and progressive exercise program is recommended for most sedentary people with T2DM to minimize the risk of injury and complications and to better adjust insulin requirements.^92^

### 6.6 What should be done when a patient has an illness?

Patients should be advised on how to manage insulin treatment during illness. Insulin dose may need to be increased by 10%–20%, depending on further blood glucose levels.^89^ In order to avoid hypoglycaemia, patients with gastrointestinal upset who are not eating, but feel well enough to continue their usual activities, may need to reduce their insulin dose.^89^ The underlying causes of the illness should be identified and treated, and insulin dose should be constantly reviewed.^89^

### 6.7 What advice should be given to patients who are going for medical procedures?

Good perioperative glycaemic control can reduce the risk of complications postoperatively in patients with diabetes.^31^ Patients should continue their insulin therapy and it has been suggested that basal insulin dose should be reduced by approximately 25% of the normal dose, the evening before surgery.^31^ The American Diabetes Association and the American Association of Clinical Endocrinologists recommend a target glucose in the perioperative period between 140 and 180 mg/dl (7.8 to 10.0 mmol/L) for critically ill patients and less than 140 mg/dl (7.8 mmol/L) for non‐critically ill patients.^31^

### 6.8 Do I need to worry about carbohydrate counting for my patients if they are only on basal insulin?

Most patients on basal insulin do not need to count carbohydrates. This is a more critical issue for patients trying to fine‐tune their prandial insulin intake to their carbohydrate intake for each meal. Focusing on this overly complicates matters for the provider and patient if only basal insulin is on board.

## 7 CONCLUSIONS

Insulin initiation should be a shared decision and follow a patient‐centred approach. Patients will need education and support to ensure success with insulin therapy. Glycaemic targets and titration strategies should be individualized. The simplest algorithm for patient and healthcare provider should be chosen and should be based on individual needs and lifestyle. It is critical patients understand insulin titration takes time and a higher dose may be required to achieve their target. Patients and providers should remain in frequent contact to ensure successful basal insulin titration. At some point, once‐daily basal insulin may no longer be sufficient and treatment intensification may be necessary.

Combination therapies of basal insulin with non‐insulin anti‐hyperglycaemic medications may result in complementary effects, including potential reductions in weight, hypoglycaemia, insulin dose, and HbA1c levels. The choice of combination therapy should be individualized based on patient characteristics and careful consideration of the advantages and disadvantages of anti‐hyperglycaemic agents. Dose adjustments and increased frequency of blood glucose monitoring may be required when co‐administrating anti‐hyperglycaemic agents with basal insulin.

Providers should address potential barriers to insulin initiation and provide reassurance that severe hypoglycaemia is rare in patients with T2DM initiating insulin. Understanding and addressing feelings and concerns about hypoglycaemia before and during treatment is critical. It is important for patients to understand the potential causes of hypoglycaemia. Modest weight gain is expected with initiation of basal insulin therapy and patient expectations need to be managed. Weight gain can be mitigated through lifestyle changes and use of combination therapies. In certain situations, for example, illness, travel, and exercise, additional monitoring, and insulin dose adjustments may be required.

In conclusion, this review was created to help address the common challenges of insulin initiation faced by patients and healthcare providers, to help optimize the use of basal insulin, and thereby improve glycaemic control in insulin‐naïve patients with T2DM.

## CONFLICT OF INTEREST

Thomas Forst has served on speaker panels for Abbott, Astra Zeneca, Böhringer Ingelheim, Berlin Chemie, Cipla, Eli Lilly, Fortbildungskolleg, MSD, Novartis, Novo Nordisk, and Sanofi; has served on advisory panels for Astra Zeneca, Bayer, Cipla, Eli Lilly, Fortbildungskolleg, Novo Nordisk, Pfizer, Sanofi, Bayer, Roche, and Eyesense. Pratik Choudhary has received speaker fees or travel support from Sanofi, Eli Lilly, Novo Nordisk, Astra Zeneca, Novartis, Insulet, Roche, Medtronic, Abbott, and Dexcom; has served on advisory boards for Medtronic, Roche, Abbott, Novo Nordisk, Lilly, Sanofi, and Insulet. Doron Schneider is a consultant and speaker for Novo Nordisk; is a consultant for Lilly and Healthcast. Bruno Linetzky is an employee of Eli Lilly and Company. Paolo Pozzilli has received research funds and travel grants from Sanofi, Eli Lilly, Novo Nordisk, Astra Zeneca, GSK, and Medtronic; has served on advisory boards for Astra Zeneca, Eli Lilly, Sanofi, and Merck.

## ETHICS STATEMENT

The ethics statement is not applicable to this manuscript as this is a review of the literature and no research subjects were involved.

## AUTHOR CONTRIBUTIONS

All authors contributed to the conception and design of the manuscript, interpretation of data, revision of the manuscript, and have read and approved the final manuscript.

8

## Data Availability

The data availability statement is not applicable to this manuscript as this is a review of the literature and no data were generated/analysed.
